# The Phenomenon of Drug Emulsion Carriers Compaction during Their Movement in Microstructures

**DOI:** 10.3390/pharmaceutics14030585

**Published:** 2022-03-08

**Authors:** Mariola M. Błaszczyk, Jerzy Sęk, Łukasz Przybysz

**Affiliations:** 1 Department of Chemical Engineering, Faculty of Process and Environmental Engineering, Lodz University of Technology, 213 Wolczanska St., 90-924 Lodz, Poland; jerzy.sek@p.lodz.pl (J.S.); luk.przybysz@o2.pl (Ł.P.); 2 Department of Refrigeration Technology and Technique in Lodz, Institute of Agriculture and Food Biotechnology, 84 Al. Marszałka J. Piłsudskiego, 92-202 Lodz, Poland

**Keywords:** emulsion carriers, drug delivery, microfluidics, capillaries, emulsion flow

## Abstract

The greatest challenges of modern pharmacology are the design of drugs with the highest possible efficacy of an active substance and with the lowest possible invasiveness for the whole organism. A good solution features the application of a bioactive substance in different carriers. The effectiveness of such preparations is determined not only by the properties of the drug, but primarily by the dynamics of carrier movement in the body. This is the reason why studies on the dispersed systems transport in micro- and nanostructures are becoming important. This paper presents a study of emulsion systems transport in microcapillaries. A dispersed phase thickening effect was observed during the process, which resulted in a concentration increase of the flowing emulsion, in some cases up to 10 times. This phenomenon directly influences transport dynamics of such substances in microstructures and should be taken into account when designing drug parameters (concentration, release time, and action range). The effect was investigated for three different emulsions concentrations and presented quantitatively. The scales of this phenomenon occurrence at different flow conditions were investigated, and their magnitudes were modelled and described. This allows the prediction of the flow resistance in the movement of given dispersion systems, as a function of the flow rate, the emulsion parameters, and the microchannel size.

## 1. Introduction

The main goal of modern pharmacology is to design such drugs that will allow the delivery of an appropriate dose of therapeutic substances to a specific site in the body without exposing the whole organism to excessive contact with them. The transport of these substances occurs in ducts formed in biological systems. It can be main arteries and veins as well as microcapillaries with blood transport. For this reason, in recent years, there has been an increasing use of various types of active substances carriers, of which emulsion carriers are one of the simplest forms [1]. The development of formulation and nanotechnology contributes to the design of, more and more, perfect methods of producing these preparations [2,3]. This opens new horizons in the pharmaceutical field. However, in addition to the improvement of such drugs, it is important how they behave in organisms, how they move, and what kind of phenomena they undergo. The knowledge will allow a more precise design of drug parameters (concentration, release time, and action range), so it is important to also focus efforts on the flow dynamics studies of various types of dispersed systems in small structures that reflect the transport routes in the body (bloodstream, intercellular transport, etc.) [4,5]. The papers [3,6] highlight various contributions of multiphase flow studies in microcapillaries as examples related to biological and pharmaceutical applications.

Dispersion systems are understood as liquids or solids dispersed in a continuous medium, for example, emulsions or suspensions. An example of such a substance is blood, where the continuous medium is plasma and dispersed substances are blood cells and other components, e.g., lipid substances, including cholesterol [7,8]. Blood flow in the circulatory system, especially in small capillaries, should therefore be considered as the transport of dispersed systems in microchannels [9,10,11]. Thus, studies of flow in microcapillaries, including those presented in this paper, can contribute to the explanation of phenomena occurring in biological structures. Understanding the phenomena occurring during the transport of dispersive systems may therefore contribute to the prediction of the movement of substances contained in the blood or introduced in the form of drugs, which may, in turn, be useful in the diagnosis and treatment of patients suffering from arterial or venous embolism.

One route of transport is through the microchannels of dermal structures, as well as vascular capillaries [12,13]. Knowledge on these migrations can be used to design new pharmaceutical or cosmetic preparations, introduced into the human body through the skin, without the use of needles. Considering the sanitary conditions of some “third world” countries and the prevailing pandemic condition, such solutions become crucial.

**Figure 1:**
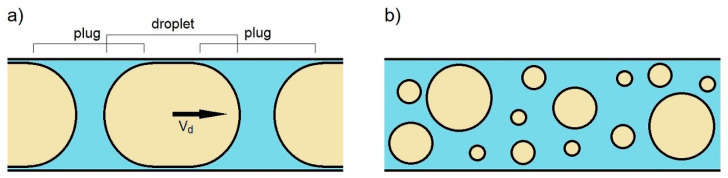
Transport of emulsions when: (**a**) the diameter of the dispersed phase is comparable in size to the channel diameter; (**b**) the average diameter of the droplets is much smaller than the channel diameter.

In the literature, the transport of dispersion systems in microchannels can be considered from two perspectives, depending on the ratio of the channel diameter to the diameter of the dispersed phase particles (droplets or particles) [14]. Considering the transport of emulsions, the first case refers to the situation in which the diameter of the dispersed phase droplets is comparable in size to the channel diameter (Figure 1a), while the second applies when the average diameter of the droplets is much smaller than the channel diameter (Figure 1b).

The first case, known as “slug flow”, is widely reported in the literature [14,15,16]. The analysis of the individual droplets transport, their deformation, and tearing in channels with a similar diameter, has gained particular interest within the formation of monodisperse emulsions, in various types of droplet generators [17,18]. The knowledge of the flow dynamics and the interactions between the phases and the channel wall proved to be extremely important for obtaining droplets of specific sizes [19]. For this purpose, capillary systems with various configurations have been used, from a simple T-shape or cross-shape to more complicated ones, which allowed obtaining single and multiple emulsions with a high degree of monodispersity. Such fluids find several applications, for example in the pharmaceutical industry as drug carriers. Due to the similar size of each droplet, it is easier to predict the rate of release of active substances placed in such carriers. However, despite the implementation of continuous improvements, the production of such liquids is relatively difficult and, above all, expensive. It is much easier and cheaper to produce polydisperse emulsions, using conventional methods [20]. However, the use of such emulsions as drug carriers involves difficulties in predicting the release rate, because the substance contained in smaller droplets may be released faster than from larger ones [2]. However, the studies on dynamics of polydisperse emulsions transport through microchannels and the development of complex models of individual droplets transport, on their basis, may provide a tool to use even such emulsions as carriers of active substances. This would allow a significant reduction in the cost of production of various types of drugs.

The transport of polydisperse systems, when the diameters of droplets or particles are smaller than the diameter of the channel (Figure 1b), has not yet been widely reported in the literature. It is generally assumed that the velocity of individual droplets is the same as the local velocity of the continuous phase. Moreover, such droplets follow the current lines of the external phase, which means that droplets closer to the channel centerline flow faster than those close to the channel walls [14,21]. The interactions between droplets and between channel walls are ignored in this approach. Such an assumption may be valid for high flow velocities, where the fluid has so much energy imparted that these interactions are of negligible importance [22]. However, for very slow flows, they become very important. The droplets can contact each other during the flow, hitting each other and the walls, and friction is then created, which means that their velocity can be lower than it would be, according to the principles of classical fluid mechanics. Because of this, there is a difference between the velocities of the continuous phase and the dispersed phase. This difference causes a very important phenomenon that occurs in such flows, namely the accumulation of droplets in the capillary structure. Droplets, of which the velocity is lower than that of the continuous phase, remain in the channel longer than the continuous phase, which flows between the droplets. As more droplets enter the channel, they do not have enough energy (velocity) to pass through the structure formed by the other droplets and begin to accumulate in it. Over time, it turns out that there are many more droplets in the channel than the initial concentration would suggest. These phenomena have not yet been captured quantitatively in the literature, so this paper undertakes to describe the phenomenon. It is traced when the phenomenon of droplet accumulation (concentration) occurs and how it depends on flow conditions.

## 2. Research Apparatus and Media

**Figure 2:**
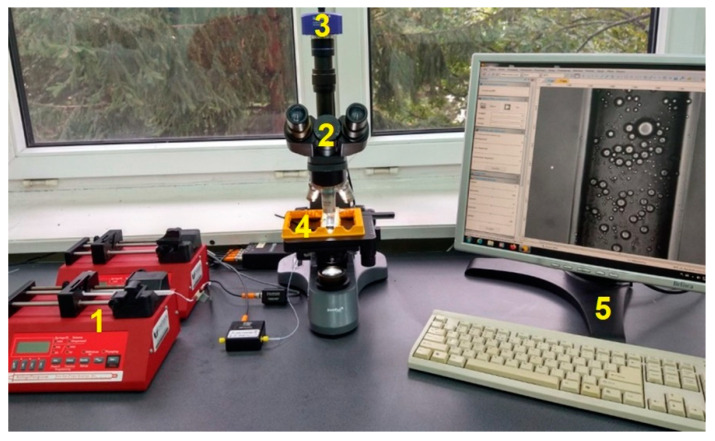
Photo of the test stand.

The study of emulsion transport through microchannels was carried out using a test stand, a photograph of which is presented in Figure 2.

The test stand consisted of a SyringeONE syringe pump (1), a LEVENHUK 740T microscope (2) with an M1000 PLUS digital camera (3), chips with hollow microcapillaries (4), and a PC (5) for data acquisition. Chips used during the research (produced by Microfluidic ChipShop) were made of poly(methyl methacrylate) (PMMA). The straight channel through which the flow took place had an equivalent diameter of 100 μm and a length of 18 mm. Measurements were made at half of the channel depth. The study of the emulsion transport consisted in forcing the emulsion with known parameters into the channel at a given flow rate using a syringe pump. The liquid was pumped, and after 30 min, the flow recording started with a digital camera. This made it possible to obtain microscopic images, which were then analyzed to determine what the actual density of oil droplets was under given flow conditions. The microscopic image analysis software SigmaScanPro 5 and LevenhukLite were used for this purpose.

Emulsions with 3 different volumetric concentrations (2.5%, 5%, and 10%) at five different flow rates (5, 10, 20, 40, and 80 μL/h) were tested. The emulsions used during the tests were an oil-in-water system, where distilled water was the continuous phase and the dispersed phase was vegetable oil with a viscosity of 60 mPa·s and a density of 892 kg/m^3^. The emulsifier used was a non-ionic surfactant with the trade name ROKACET 07 (PCC Exol SA). Its role was to ensure the full stability of the research media used, i.e., emulsions, for the duration of the experiments, which was a key condition for the conducted research. The 10% emulsions were prepared by mixing the appropriate volumes of oil and water with a 2% volume fraction of the emulsifier. Emulsification was carried out for 300 s using a high-speed homogenizer providing a rotation speed of 20,000 rpm.

In order to avoid changes in the distributions of the oil droplets diameter, emulsions with concentrations of 5% and 2.5% were made by diluting the base 10% emulsion with distilled water. The produced emulsions were then analyzed in terms of their properties and stability. The measurements of the investigated liquids viscosity were carried out using a shear rheometers Bohlin CVO-120 (Malvern Instruments, Malvern, UK). The stability was measured by using an optical analyzer TurbiscanTM LAB (Formulation Inc., Toulouse, France). All emulsions prepared behaved as Newtonian liquids, and the viscosities of the 10%, 5%, and 2.5% emulsions were 2.67 ± 0.5, 1.94 ± 0.5, and 1.35 ± 0.5 mPa·s, respectively. The optical tests of the formed emulsions with the use of TurbiscanTM LAB for 180 min showed no changes in the emulsion structure, which means their stability during the study of the flow process through the microcapillaries.

## 3. Results

**Figure 3:**
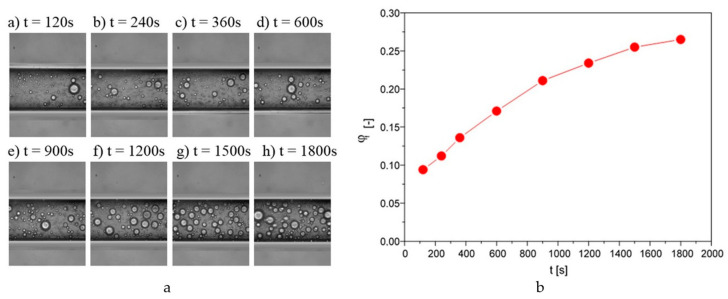
(**a**) Sequence of images taken during the flow of an emulsion with a concentration of 0.05 at a flow rate of 5 μL/h. (**b**) Changes in the droplet density ratio during the flow through the capillary for the emulsion with a concentration of 0.05 at a flow rate of 5 μL/h.

By tracking the flow of a polydisperse emulsion through a straight channel, it was noted that droplet accumulation occurred over time. To capture this phenomenon quantitatively, microscopic images were recorded overtime, after the flow was initiated. A sequence of such images for an emulsion with an initial concentration of 0.05 at a flow rate of 5 μL/h is shown in Figure 3a.

As can be observed from the presented images in Figure 3a, the number of droplets inside the capillary increased with time. Based on microscopic image analysis, it was possible to determine what fraction was occupied by oil droplets in relation to the total. This allowed determining parameter *φ_f_* further referred to as the density parameter. This parameter can be a certain indicator of what concentration of the internal phase of the emulsion the flow takes place with under given conditions. As it turns out, for the analyzed case, the value of this parameter as early as after two minutes of the flow was 0.094, i.e., the concentration of the internal phase of the emulsion increased almost twice in relation to the initial concentration (0.05), which can be seen in Figure 3b. With time, there was a further increase in the compaction ratio. However, after some time, the observed changes were already negligible, and it could be assumed that a further flow took place at a constant droplet concentration, i.e., a steady state was reached.

The dynamics of the oil droplet density changes during a flow were not the focus of this work. It was decided to focus on the steady state, i.e., the state in which there were no further significant temporal changes of the concentration degree. It was assumed that such a state was reached in the case of the presented research after 30 min. This allowed determining how the degree of compaction of the emulsion oil droplets depended on the flow conditions and the initial concentration of the emulsion. 

**Figure 4:**
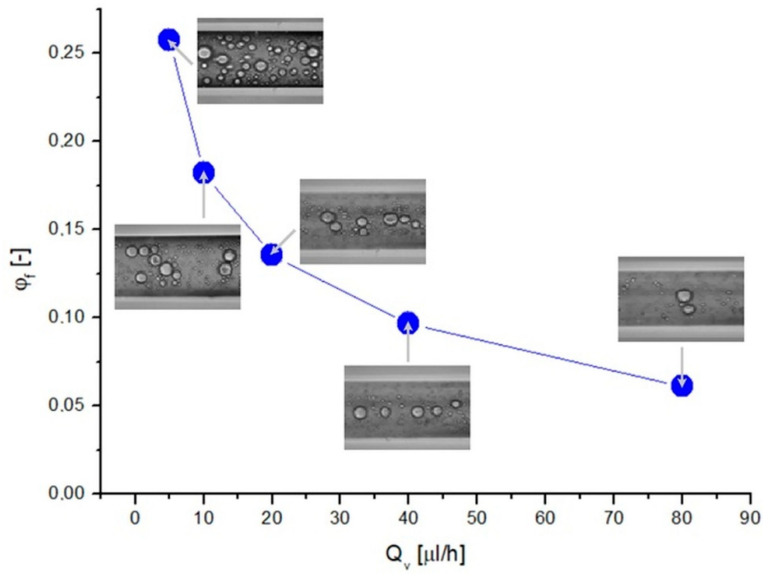
Dependence of the degree of compaction of the emulsion with a concentration of 0.05 on the liquid flow rate.

The intensity of the droplet aggregation phenomenon depended on the liquid flow rate. The emulsion flow through the capillary was tested at five different flow rates for three initial concentrations of the emulsion. The emulsions were allowed to flow for a predetermined time until a steady state was reached (30 min), and after this time, it was examined how much of the capillary was occupied by oil droplets and how much was occupied by the continuous phase at a given section of the tube. To do this, microscopic images obtained during the measurements were analyzed. The dependence of the compaction ratio on the flow rate for the emulsion with a volume fraction of the internal phase equal to *φ* = 0.05, along with the corresponding microscopic images, are presented in Figure 4.

By analyzing the change in the droplet density for increasing liquid flow rates, it can be observed that the value of *φ_f_* decreased. Even from visual assessment alone, based on microscopic images, it can be seen that at low flow rates, the number of oil droplets was definitely higher than for flows with greater flow rates. In addition, the large droplets showed a tendency to the flow closer to the channel axis, which was especially noticeable at a high fluid flow rate (*Q_v_*). The value of the compaction ratio was, for low flow rates, many times greater than the initial concentration value (*φ* = 0.05). This indicated the occurrence of the compaction phenomenon, which means that the number and volume of oil droplets in the capillary structure were larger than those of the droplets in the emulsion before entering the structure. As the flow rate increased, the degree of compaction decreased and, for high values of *Q_v_*, reached a value close to the value of the initial concentration of the emulsion. This means that the compaction phenomenon occurred more intensively when the liquid flow rate was lower. 

**Figure 5:**
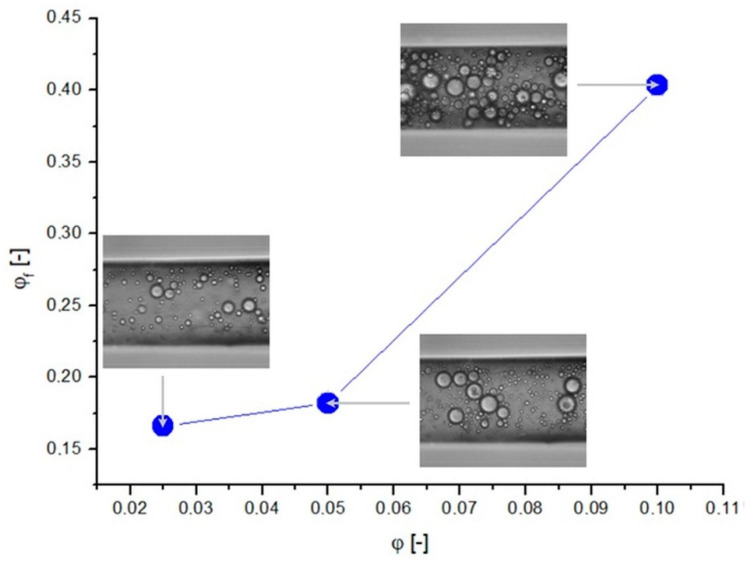
Dependence of the degree of compaction on the initial concentration of emulsion at a liquid flow rate of 10 μL/h.

For comparison, Figure 5 also shows graphically the dependence of the compaction rate on the initial emulsion concentration with the corresponding microscopy images (*Q_v_* = 10 μL/h).

The largest number of oil droplets could obviously be observed for the emulsion with the highest concentration (*φ* = 0.1; see Figure 5), where the oil droplets occupied most of the capillary. As a result of the thickening phenomenon, the oil droplets were in contact with each other and directly affected the flow of neighboring droplets. Droplets closer to the center of the channel having a higher velocity, hooking the droplets closer to the wall, could pull them with each other and give them a higher velocity. On the other hand, the droplets that were closer to the wall, being in contact with the droplets closer to the center of the duct, could decelerate them. In such a situation, the flow became more complicated, and ignoring the interaction between the droplets and assuming that their velocity depended only on the local velocity vectors of the continuous fluid would result in serious errors in the transport estimation of such systems. Therefore, a careful analysis of the incidence of droplet compaction on flow conditions is necessary.

**Analysis of Mean Values of the Compaction Changes**

Knowing the fluid flow rate (*Q_v_*), channel dimensions (*d_z_*), and continuous fluid parameters (*η_c_* and *ρ_c_*), it was possible to calculate the Reynolds number according to the relationship:

(1) $$Re=\frac{u\cdot d_{z}\cdot \rho_{c}}{\eta_{c}},$$

where *u* is the velocity of the fluid flow calculated from the relation, *u = Q_v_*/*A_p_* (where *A_p_* is the cross-sectional area), *d_z_* is the channel equivalent diameter, and *ρ_c_* and *η_c_* are the density and viscosity of the continuous phase, respectively.

**Figure 6:**
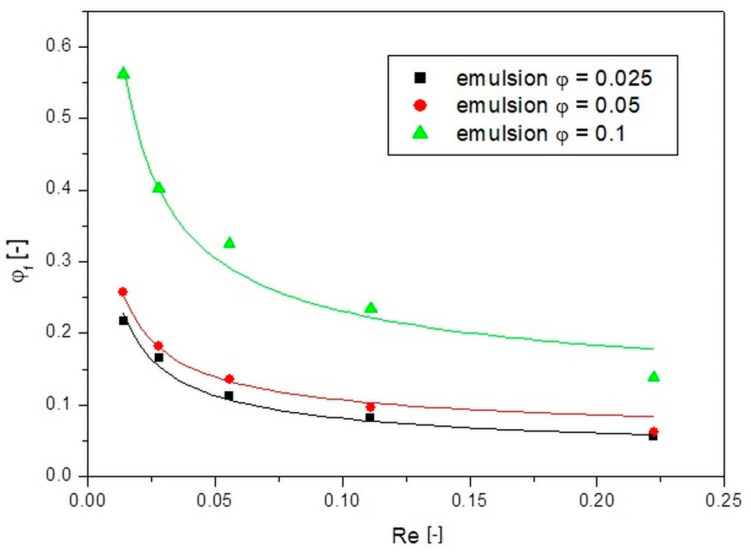
Dependences of the compaction ratio on the Reynolds number for different emulsion concentrations.

This allowed plotting the dependence of the compaction factor on the Reynolds number value, which is shown in Figure 6 for the studied emulsions. 

From the results presented in Figure 6, it is possible to quantitatively trace what the actual compaction of the emulsion oil droplets was during a flow through the channel. It turned out that, for small Re numbers and thus small flow rates, the value of the compaction factor was many times greater than the value of the concentration of the emulsion that was introduced into the channel. By dividing the value of the compaction factor *φ_f_* by the value of the concentration of the emulsion being introduced *φ*, it can be easily calculated how many times the concentration of emulsion in the channel increased. For the lowest flow velocities tested, at the lowest Re for an emulsion with an initial concentration of 0.025, the value of the actual concentration was as much as nearly 10 times the value of the initial emulsion concentration, while for an emulsion with an initial concentration of 0.1 it was five times greater. This, therefore, confirmed the visual inference that at low emulsion flow rates inside the capillary there was a thickening of the emulsion oil droplets. This is because the continuous phase did not have enough energy to entrain the droplets with themselves, so they flowed at a lower velocity than the continuous phase, resulting in their increased number in the channel over time. As the Re number (i.e., fluid flow rate) increased, the fluid energy increased, and the effect of this compaction decreased. For the highest Re values, the compaction factor values were close to the initial emulsion concentration (the concentration of emulsion entering the channel). Two facts can be concluded from these analyses. The first is that the actual concentration of the emulsion flowing through the capillary was higher than the concentration of the emulsion being introduced. The second is that this actual concentration was closely related to the imparted flow velocity. Finding a quantitative relationship between the parameters is another challenge posed when designing a drug carrier transport into capillary structures (capillaries, intercellular cement, etc.). 

It should be noted, however, that the thickening phenomenon occurred only at very low Reynolds number values. In such a situation, the energy of the fluid was so low that it was not able to carry away the oil droplets of the emulsion. The oil droplets, as a result of interactions (rubbing against each other, bumping against each other, etc.) and due to the contact with the capillary walls, lost their velocity. Such droplets started to accumulate in the capillary structure, while the continuous phase flowed through them as through a sieve. However, at higher Reynolds numbers, this phenomenon did not occur. The energy of the fluid was so high that the oil droplets did not accumulate in the capillary structure but flowed together with the continuous phase. Therefore, it can be concluded that there is a certain limiting value of the Reynolds number, beyond which the effect of droplet aggregation does not occur. Based on the research presented here, it can be determined that this limiting Reynolds number value, denoted as Re_k_, was 0.22. Above this value, the compaction factor recorded after 30 min of flow reached a value close to the concentration of the emulsion entering the capillary. The determination of such a critical value of the Re_k_ number may be crucial in the analysis of microfluidic dispersion systems at very low velocities such as occurring in capillaries or in the intercellular cement of the skin. This criterion can be a useful tool to quickly assess whether, for a given case of a fluid motion, one should consider changes in the nature of the flow associated with the phenomenon of dispersed phase compaction or whether this phenomenon can be neglected and the flow can be considered as homophasic.

If it is determined that, for the conditions under study, the calculated Re number is less than the critical value of Re_k_, then it is known that the compaction phenomenon can occur. However, the question remains on what scale it occurs. To determine this, it is useful to know the dependence of the compaction ratio on the Reynolds number. To describe this relationship, an equation of the following form was used:

(2) $$\varphi_{f}=\frac{a}{Re^{b}}+c,$$

where *φ_f_* is the compaction factor [-], *a* is the variable equation parameter, *b* is a constant equation parameter equal to 0.65, and *c* is the initial concentration of the emulsion (*c* = *φ*).

The graph marked as Figure 6 shows the fit of the presented model to the experimental data as continuous lines. The model has three parameters, with parameter c corresponding to the initial concentration of the emulsion and parameter *b* taken as a constant for the presented data, which was 0.65. The summary of the parameter a along with the coefficient of determination R^2^ is shown in Table 1.

As can be seen, the proposed relationship described the experimental data well and can be used to predict the emulsion compaction phenomenon in channels. It is worth noting that for large values of Re numbers, according to this model, the value of *φ_f_* took the values of the initial concentration *φ*, i.e., there was a lack of compaction phenomenon, which is consistent with the experiment.

**Analysis of Local (Zonal) Compaction Changes in the Capillary**

Due to the fact that the phenomenon of droplet compaction occurred to a different extent depending on their locations in the channel, in the later part of this work, an analysis of the degree of emulsion compaction under given flow conditions, but depending on the distance from the center of the channel, was undertaken. The concept of dividing the capillary into three zones was adopted. The first zone was closest to the channel center, the second zone was the intermediate zone, and the third zone was closest to the channel wall. The sizes of these zones were equal to each other. Given that the width of the channel was 100 μm, the limits of the first zone from the channel axis were ±16.7 μm. The second zone was between ±16.7 and ±33.3 μm from the axis, while the third zone was between ±33.3 and 50 μm from the axis.

**Figure 7:**
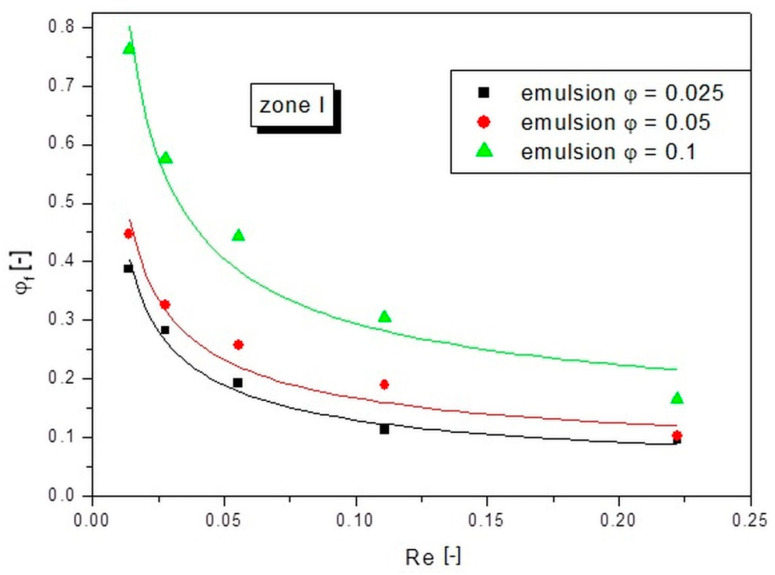
Dependences of the actual concentration on the Re number of the liquid for different emulsion concentrations in zone I.

In order to better illustrate the described compaction phenomenon, plots of the dependence of the change in actual concentration on the Re number for the three zones are separately presented. This helped to show how the initial concentration of the emulsion affected the scale of compaction. Figure 7 shows the plot of the Reynolds number versus the actual concentration of the emulsion in zone I, concerning droplets situated closest to the capillary axis. 

As can be seen from the results presented in Figure 7, the values of the actual concentrations far exceeded those of the emulsion entering the capillary. The more concentrated the input emulsion, the higher the concentration values of *φ_f_*. However, for the emulsions with *φ* = 0.025 and *φ* = 0.05, the values were more similar to each other than compared to the *φ* = 0.1 emulsion. The compaction phenomenon occurred more intensely at low flow rates. The emulsion with *φ* = 0.1 at the lowest set flow rate (lowest Re number) underwent eightfold (counting *φ_f_*/*φ*) compaction in zone I. For the emulsion with an initial concentration of *φ* = 0.05, the compaction was ninefold under the same conditions, whereas for the emulsion with *φ* = 0.005 the compaction was as much as 15-fold. The smaller increase in the actual concentration for emulsions with higher initial concentrations can be explained by the fact that a higher concentration led to the generation of more droplets and the more droplets were able to form more compact structures. The flowing continuous phase was unable to bypass these compact structures, so the two phases flowed together. For emulsions with low initial concentrations, the continuous phase had plenty of space around the droplets and could easily bypass them. The continuous phase flowed between the droplets, while the droplets flowed slower due to the resistances from the interaction between them and between them and the walls. These differences in the flow velocity between the continuous phase and the dispersed phase caused a large increase in the concentration ratio. Therefore, it can be concluded that the actual concentration (compaction) of the droplets was dependent on both the fluid flow conditions and the initial concentration of the emulsion. 

**Figure 8:**
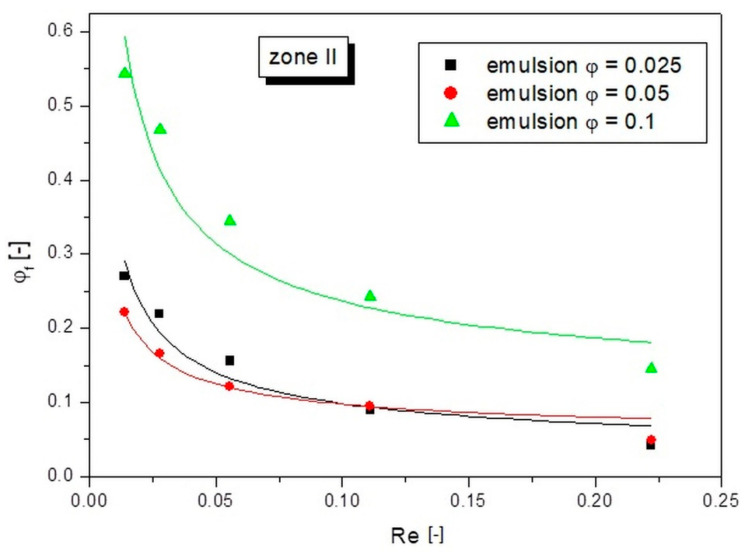
Dependences of the actual concentration on the Re number of the liquid for different emulsion concentrations in zone II.

Figure 8 shows the analogous relations obtained for zone II, i.e., intermediate zone. The effect of compaction was smaller here than in the case of zone I, but the gained results allowed making the following conclusions. 

In zone II, the greatest increase in compaction also occurred at the lowest Re values. In this case, the emulsion with an initial concentration of *φ* = 0.1 reached after 30 min of the flow, the compaction value *φ_f_* was more than twice as high as the initial concentration. For the emulsion with an initial concentration of *φ* = 0.05, the compaction value *φ_f_* was more than four times, while the compaction value *φ_f_* was more than 21 times for the emulsion with an initial concentration of *φ* = 0.025. This means that for these lowest initial concentrations of emulsions in zone II, even greater compaction occurred than in zone I. This is due to the fact that in zone II, due to the symmetrical velocity profile, the energy of the liquid was lower than in zone I. When the energy of the fluid was smaller, the flow resistance began to play a larger role. The fluid did not have enough energy to overcome the flow resistance of the oil droplets, so there was a deceleration of the velocity of the droplets and consequently an increase in their density in the structure. Finally, the values of *φ_f_* for the emulsion with an initial concentration *φ* = 0.025 even exceeded the values obtained for the emulsion with an initial concentration *φ* = 0.05. 

**Figure 9:**
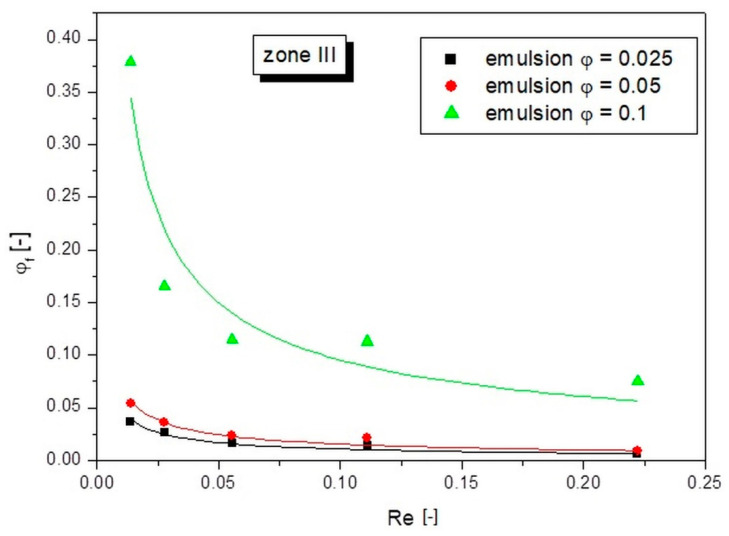
Dependences of the actual concentration on the Re number of the liquid for different emulsion concentrations in zone III.

From the perspective of the transport of polydisperse emulsions through capillary structures, of great importance is how the values of the actual concentrations were formed in zone III, which was the one closest to the wall. The results obtained for this zone are presented in Figure 9. 

Based on the results obtained for zone III, it can be seen that the largest changes were recorded for the emulsion with an initial concentration *φ* = 0.1. Analogous to the previous cases, with the lowest set flow value (lowest Re number), the recorded *φ_f_* values of the actual concentration for the emulsions with concentrations *φ* = 0.05 and *φ* = 0.025 were at the level of the initial concentration. However, for the higher concentrated emulsion (*φ* = 0.1), there was still a compaction effect, and the *φ_f_* values increased by more than 3.5 times *φ*. This means that at higher initial emulsion concentrations, there was such a high density of oil droplets that they occupied the bulk of the capillary volume. The presence of oil droplets near the wall increased the friction with the wall, which in turn affected the fluid flow velocity. On the other hand, the emulsion thickened throughout the volume formed a compact structure. The flow of such a structure should be considered as a whole. As written earlier, oil droplets closer to the center (with a higher average velocity) interlocked with droplets closer to the wall giving them a higher velocity. In turn, those that were closer to the wall decelerated those that were closer to the center. 

Equation (2) was used to describe the dependence of the compaction factor on the Reynolds number for each zone. The results of fitting the calculated points with this equation to the experimental points are presented in Figure 6, Figure 7, Figure 8 and Figure 9 as continuous lines. The values of parameter a and the obtained determination coefficients R^2^ are shown in Table 2. The value of parameter b was 0.65 in each case, while the values of parameter c for zones I and II were set as the value of the initial emulsion concentration *φ*. However, in the case of zone III, taking parameter c as the initial emulsion concentration would not be valid, because this zone had the fewest oil droplets and its concentration was below the initial emulsion concentration. Therefore, a value of parameter c of 0 was arbitrarily adopted for zone III, which resulted in a good fit to the experimental points for all emulsion flows tested. 

## 4. Summary and Conclusions

Based on the work carried out, it was found that under certain flow conditions, there was a phenomenon of accumulation of oil droplets in a channel structure. These conditions depended on the flow rate, fluid parameters, and flow geometry. It was concluded that the compaction phenomenon is due to the fact that the oil droplets flowing through the channel collided with each other or with the channel walls and, as a result, their velocity was decelerated. The continuous phase, on the other hand, flowed through the channel, bypassing the oil droplets. As a result, the oil droplets accumulated in the channel structure, and after some time, their quantity was much higher than the original. It was determined that the droplet concentration phenomenon became significant when the value of the Re number exceeded the value of the Re_k_ number equal to 0.22.

Comparing the state of droplet accumulation after the same period of time (from the beginning of the flow), it can be observed that the lower the liquid flow rate was, the higher the compaction phenomenon was. Moreover, comparing the flows of emulsions with different concentrations, it was observed that for the emulsions with the lowest concentrations the degree of compaction increased even several dozen times in relation to the original compaction (initial concentration of the emulsion). The dependence of the degree of compaction on the Reynolds number has been described by a model dependence, which allowed estimating the scale of the phenomenon under given flow conditions. In addition, the study presents an analysis of the degree of compaction of droplets as a function of their distance from the pipe axis. It was observed that the degree of compaction in particular zones depended on the initial concentration of the emulsion, especially for the zone closest to the wall (zone III). The dependences of the compaction ratio on the Reynolds number for individual zones were also described by the model dependence shown as Equation (2). 

The awareness of the occurrence and magnitude of the dispersed phase compaction phenomenon and the ability to accurately predict it can be extremely important in many practical aspects, for example, in monitoring blood flow, especially in small capillaries where flow velocities are low. In such situations, the concentration of blood cells or other blood constituents such as cholesterol may be much higher than indicated by laboratory analysis. Quantitatively capturing the degree of compaction under given conditions, therefore, may be useful in counteracting venous congestion and cholesterol deposition. Another practical use of studies on dispersion compaction at low flow rates may be to predict the permeation of micro- and nanoemulsions through various porous structures such as oil rocks or skin layers.

## Figures and Tables

**Table 1:** Summary of parameter a and the coefficient of determination.

Initial Concentration of the Emulsion *φ* [-]	*a*	R^2^
0.025	0.01254	0.982
0.05	0.01269	0.974
0.1	0.02927	0.968

**Table 2:** Summary of the parameters in Equation (2) for the three emulsion flow zones.

	a	b	c	R^2^
Zone I				
Emulsion with an initial concentration of 2.5%	0.02342	0.65	0.025	0.985
Emulsion with an initial concentration of 5%	0.02613	0.65	0.05	0.954
Emulsion with an initial concentration of 10%	0.04359	0.65	0.1	0.959
Zone II				
Emulsion with an initial concentration of 2.5%	0.01648	0.65	0.025	0.932
Emulsion with an initial concentration of 5%	0.0107	0.65	0.05	0.951
Emulsion with an initial concentration of 10%	0.0306	0.65	0.1	0.916
Zone III				
Emulsion with an initial concentration of 2.5%	0.00241	0.65	0	0.961
Emulsion with an initial concentration of 5%	0.00349	0.65	0	0.958
Emulsion with an initial concentration of 10%	0.02133	0.65	0	0.905

## Data Availability

Not applicable.
